# Analysis of Intestinal Short-Chain Fatty Acid Metabolism Profile After Probiotics and GLP-1 Treatment for Type 2 Diabetes Mellitus

**DOI:** 10.3389/fendo.2022.892127

**Published:** 2022-06-30

**Authors:** Qiuxia Min, Yan Wang, TaiCheng Jin, Lei Zhu, XianYan Wu, YiKun Li, YanJiao Wang, Ning Xu

**Affiliations:** ^1^ Department of Pharmacy, The First People's Hospital of Yunnan Province, Kunming City, China; ^2^ Department of Endocrinology, The First People’s Hospital of Yunnan Province, Kunming City, China; ^3^ Hamilton College, NY, United States; ^4^ Department of Clinical Laboratory, The First People's Hospital of Yunnan Province, Kunming City, China

**Keywords:** probiotics, GLP-1, type 2 diabetes, short-chain fatty acid, metabonomics

## Abstract

Type 2 diabetes accounts for about 90% of diabetes patients, and the incidence of diabetes is on the rise as people’s lifestyles change. Compared with GLP-1 treatment, probiotic treatment can directly regulate homeostasis of the host gut microbe, and thus homeostasis of its metabolites. Currently, the regulatory role of probiotics on intestinal metabolites after treatment of type 2 diabetes mellitus remains unclear. The purpose of this study was to investigate the therapeutic effect of probiotics on type 2 diabetes mellitus and its regulatory effect on short-chain fatty acids, which are metabolites of intestinal microorganisms. I collected feces from 15 patients with diabetes before treatment and 15 patients with type 2 diabetes after treatment with GLP-1 and probiotics. The abundance of short-chain fatty acids in feces was determined by GC-MS. Results Both GLP-1 and probiotics could improve the levels of blood glucose, urine glucose and BMI in patients with type 2 diabetes. After glP-1 treatment, two short-chain fatty acids (butyric acid and valerate acid) in intestine were significantly changed. Propionic acid and isovalerate were significantly changed after probiotic treatment. At the same time, KEGG signal pathway enrichment results showed that probiotics intervention mainly achieved the purpose of treating type 2 diabetes through regulating protein and carbohydrate metabolism. Taken together, our study shows changes in intestinal short-chain fatty acids after probiotics or GLP-1 treatment of type 2 diabetes, which will provide us with new insights into the mechanism of probiotics treatment of type 2 diabetes, as well as potential intervention targets for diabetes treatment.

## Introduction

According to statistics, type 2 diabetes (T2DM) patients account for about 90% of all diabetes cases (1). In China, the proportion of the elderly population will rise to 18.7% in 2020, and the incidence of diabetes among the elderly is 30%, 95% of which are type 2 diabetes (T2DM) (2). The human gut is home to more than 1,014 species of microbes, known as the gut microbiome (3). In recent years, more and more studies have been conducted on the relationship between intestinal microbes and the occurrence of diseases (4). Biomedical studies have shown that the occurrence of type 2 diabetes is related to the human intestinal microbiota. Intestinal microecological, also contains a large number of gut microbes metabolites in the disease occurrence and progress of gut microbes metabolites is usually gut microbes interact with the host medium, the metabolites can affect the host immune and can be directly participate in the process of cell biology into the circulatory system, therefore, the normal intestinal flora is very important for the stability of the intestinal environment (5, 6).

In healthy people, intestinal L-cells secrete glucagon-like peptide-1 (GLP-1) after eating, which reduces glucose concentration by increasing insulin secretion and inhibiting glucagon release (7). At present, many GLP-1-related drugs have been developed, which can be divided into short-acting compounds and long-acting compounds. Short-acting compounds such as exenatide and Lixisenatide can activate GLP-1 receptor in a short time and reduce postprandial blood glucose levels mainly by inhibiting gastric empting (8, 9). Long-acting compounds such as Albiglutide and dulaglutide can activate the GLP-1 receptor for long time and control fasting glucose mainly by Raising insulin levels and lowering glucagon levels (10). Although these drugs have been widely used in the clinical control of diabetes, the use of glP-1 receptor-associated drugs in some populations has been associated with severe gastrointestinal reactions (nausea, diarrhea, and vomiting) and even anorexia (11–13). Therefore, it is still necessary to find more mild and effective methods for the treatment of diabetic patients.

SCFA is a subset of fatty acids produced by the gut microbiota during the fermentation of indigestible polysaccharides (14). The association of plasma and colonic SCFA with metabolic syndrome has been well documented. Similarly, SCFAs play an important role in the regulation of energy homeostasis, and thus have potential as therapeutic targets for metabolic diseases, such as obesity and type 2 diabetes (15). In terms of the mechanism of action, the production of microbial SCFA is crucial to the integrity of the intestine, which can affect mucosal immune function by regulating the pH value in the intestinal lumen, the secretion of intestinal mucus and the nutrient absorption of epithelial cells (16, 17). SCFA also directly regulates host metabolic homeostasis through several tissue-specific mechanisms related to appetite regulation, energy consumption, glucose homeostasis and immune regulation (18, 19). Therefore, the homeostasis of short-chain fatty acids (SCFA), a metabolic product of intestinal microorganisms, plays a crucial role in the occurrence and development of type 2 diabetes mellitus, and can serve as a marker and therapeutic target for metabolic disorders and balance.

Currently, there are no effective reports on the treatment of type 2 diabetes with short-chain fatty acid-related preparations, but as metabolites of intestinal flora, probiotics can be directly used to regulate the chemotaxis of intestinal flora in patients with type 2 diabetes, thus regulating metabolic homeostasis of the body. Probiotic therapy has been widely known in recent years, and current studies have shown that improving the composition of intestinal microbiota can have effective and long-term benefits on body development, intestinal diseases, and metabolic diseases compared to drugs and other therapies that act on gene loci (20, 21). In addition, probiotics have been proven to have potential and related effects in the treatment of type 2 diabetes, such as improving intestinal barrier function, lowering blood glucose and increasing insulin secretion (22).

In summary, fecal samples from 15 type 2 diabetes patients and 15 type 2 diabetes patients treated with probiotics were collected for targeted metabolomics detection of short-chain fatty acids, while fecal samples from 15 type 2 diabetes patients treated with GLP-1 were collected for metabolic analysis of short-chain fatty acids.

## Materials and Methods

### Subject Recruitment and Sample Collection

To investigate the intestinal microbe short-chain fatty acid metabolism in patients with type 2 diabetes after probiotics treatment, we recruited 15 patients with type 2 diabetes from May 2021 to December 2021 in the First People’s Hospital of Yunnan Province, and treated them with probiotics (Combined Bifidobacterium, Lactobacillus, Enterococcus and Bacillus cereus Tablets, S20060010, Si Lian Kang) under the same conditions. At the same time, hBA1c, urine glucose, blood glucose, 2h postprandial glucose, body weight, BMI, visceral fat, triglyceride (TC), and total cholesterol (TG) levels were recorded in 15 patients on admission and after treatment. At the same time, to understand the changes of short-chain fatty acids in the intestines of type 2 diabetes patients after GLP-1 treatment, we collected feces from 15 type 2 diabetes patients treated with GLP-1. In order to ensure the objective situation of short-chain fatty acid metabolism and collection, the feces of the subjects were collected in a sterile test tube in the morning 8h after fasting, and quickly stored in liquid nitrogen.

### Sample Preparation

About 50mg ± 10 mg stool samples were accurately weighed. 500 μL methanol-aqueous solution (containing 0.1%HCL, 20%H2O) and isotope internal standard acetate -d4 were added to the samples. The samples were then crushed by a frozen crusher, followed by ice bath ultrasound for 10 min, vortex mixing and centrifugation to obtain supernatant (12000 rpm, 4 °C, 5 min), 200 μL of the supernatant was diluted and put into the sample bottle for gC-MS analysis

### GC - MS Analysis

In this project, Thermo Scientific TSQ 8000 Evo mass spectrometry and Trace 1310 gas chromatograph were used for chromatographic separation of target compounds by FFAP column. Conditions of GC-MS/MS, Injector temperature 280°C, Mass spectrometry transfer temperature 240°C, Ion source temperature 300°C, Initial temperature 50°C (1min), Set the temperature to rise to 180°C at the rate of 1°C/min and then to 240°C at the rate of 40°C/min (hold for 3 min, Flow rate: 1.2mL/min). The standard solution of the target compound was introduced into the mass spectrometry before mass spectrometry analysis. EI source voltage is 70eV.

### Quantitative Detection

This project was quantified by internal standard method, and the retention time and SIM fragment ions were qualitatively compared by standard method. All mass spectrometry data collection and quantitative analysis of target compounds were performed by Thermo Scientific Xcalibur. During the test, the samples whose concentration is higher than the calibration curve range are tested again after being diluted to an appropriate multiple, and the measured data after dilution are used as quantitative results. The calculated final concentration of the sample is calculated by multiplying the calculated concentration by an dilution factor. The concentration of target metabolites in the sample is equal to the mass concentration of target metabolites in the sample

### Statistical Analyses

An important part of metabonomics research is to search for biomarkers, that is, the screening of differential metabolites. In the univariate statistical analysis of this project, p value was calculated by t test to evaluate the statistical significance of differential variables. The differential Fold change of metabolite quantification was calculated. It is generally believed that p value <0.05, a Fold change>2 or < 0.5 as differential metabolism.

Principal Component Analysis (PCA) is an unsupervised multidimensional statistical Analysis method, which can reflect the overall metabolic differences among each group and the variation degree among samples within the group. In the two-dimensional graph, we take the first two principal components PC1 and PC2 as the horizontal and vertical axes to represent the sample, thus reducing the data from multidimensional to 2-dimensional. The parameter that measures the ability of the principal component to describe the characteristics of the model is called the explanatory rate. In the figure, PC1 is the explanatory rate of the first principal component, and PC2 is the explanatory rate of the second principal component. If the samples are similar, they should be shown close to each other on the diagram, which also illustrates the quality of the samples’ repeatability.

## Results

### Study Population and Clinical Parameters

**Figure 1:**
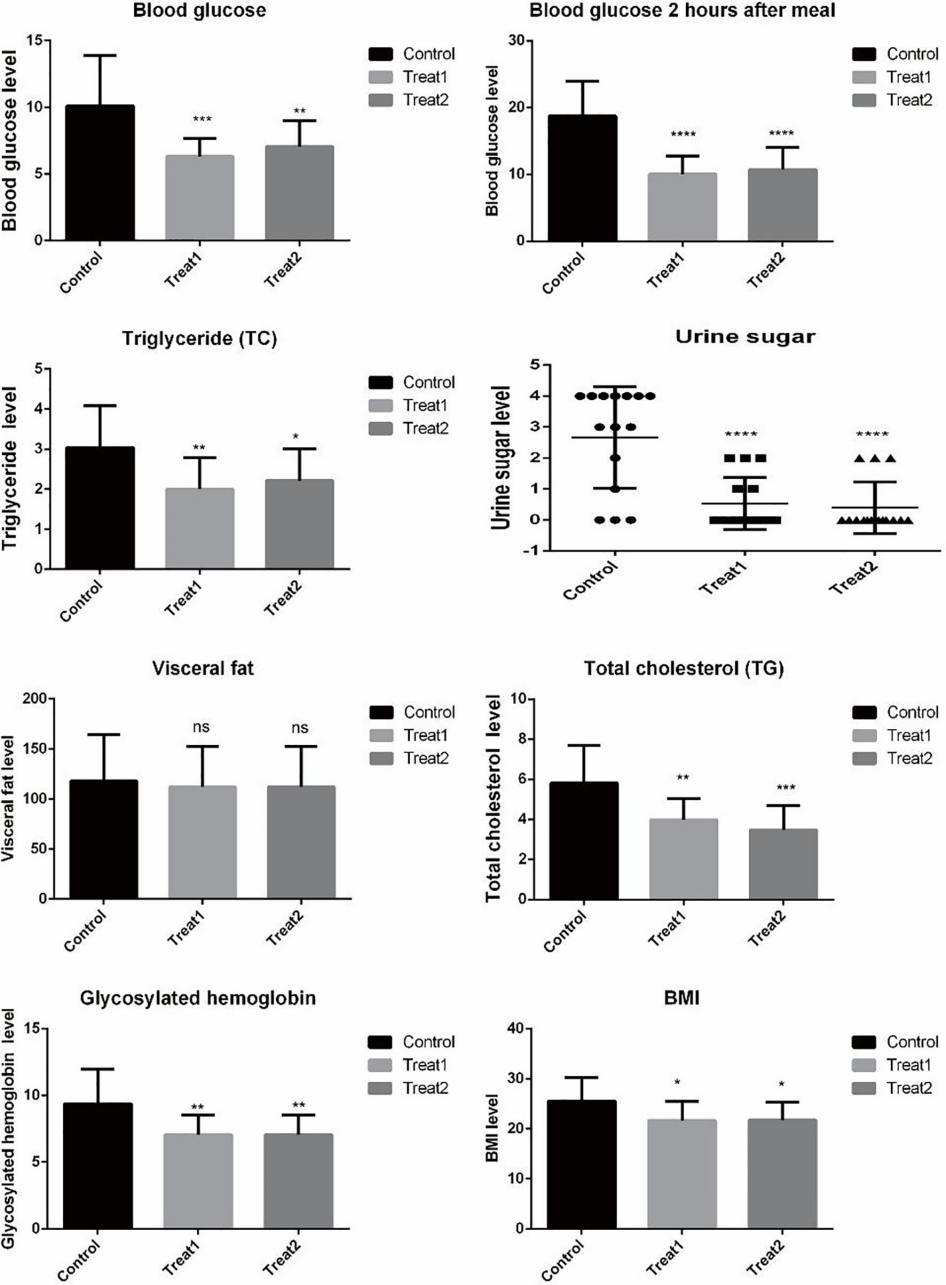
Table of biochemical indexes of patients in different groups. *, **, ***, **** are respectively compared with the control group p value< 0.05, p value<0.01, p value<0.001, p value<0.0001.ns compared with the control group P value> 0.05.

In this study, faeces from 15 diabetic patients aged 31-91 years were collected, including 11 males and 4 females. Stool samples were collected from 15 patients aged 31-91 (10 males and 5 females) treated with GLP-1 for type 2 diabetes. Stool samples were collected from 15 patients aged 31-91 (11 males and 4 females) treated with probiotics (Combined Bifidobacterium, Lactobacillus, Enterococcus and Bacillus cereus Tablets, S20060010, Si Lian Kang)for type 2 diabetes. See **Table 1** for details. To evaluate the clinical efficacy of GLP-1 and probiotics before and after treatment of type 2 diabetes, we examined the physical indicators of patients. Glycosylated hemoglobin, Blood glucose, Blood glucose 2 hours after meal, BMI, Visceral fat, Triglyceride, urine Sugar and Total cholesterol. As shown in **Figure 1**, Glycosylated hemoglobin, Blood glucose, Blood glucose 2 hours after Treat1 group and Treat2 group The levels of meal, BMI, Triglyceride and Total cholesterol were significantly reduced (P Value< 0.05). Urine sugar testing showed improvement in both probiotics and GLP-1 treatment, with more urine sugar returning to normal in the probiotics group. After treatment, there was no significant difference in visceral fat among all groups.

**Table 1:** Clinical test information.

Parameter	Control	Treat1	Treat2
gender (male/female)	11/4	10/5	11/4
Age (Years)	31-91	31-91	31-91
Glycosylated hemoglobin(%)	5.56-12.54	4.72-9.67	4.72-9.67
Blood glucose	5.1-17.4	4.1-8.9	4.4-11.9
Blood glucose 2 hours after meal	7.3-28.1	7.2-15.8	7.1-17.8
BMI	15.88-33.3	12.1-26.7	13.1-26.7
Visceral fat	40-189	41-187	41-187
Triglyceride (TC)	1.08-5.35	1.08-3.56	1.09-3.63
Total cholesterol (TG)	0.35-9.83	0.93-5.21	1.26-5.52

### Metabolic Profiling of Diabetic Patients Before and After Treatment

**Figure 2:**
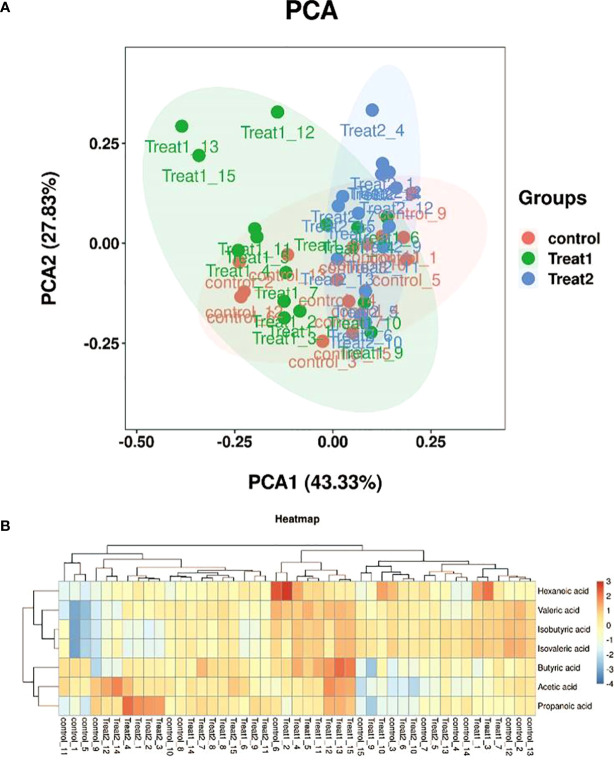
PCA plots of each group and differential expression heat maps of samples. **(A)** Principal Component Analysis (PCA) of all samples. **(B)** Heat maps of short-chain fatty acid expression in all samples.

In **Figure 2A**, PC1 in parentheses is the interpretation rate of the first principal component, and PC2 in parentheses is the interpretation rate of the second principal component. If the samples are similar, they should be shown close to each other on the diagram, which also illustrates the quality of the samples’ repeatability. In the samples of this study, the separation degree of glP-1 treating type 2 diabetes group (Treat group 1) and probiotics treating type 2 diabetes group (Treat group 2) was large, and the separation degree of Control group and Treat 1 and Treat 2 group was small. As shown in **Figure 2B**, 7 short-chain fatty acids are detected in this targeted metabolomics, which are Acetic acid, Propanoic acid, Isobutyric acid, Butyric acid, Isovaleric acid, respectively. Valeric acid and Hexanoic acid. In general, there was little change in the short-chain fatty acids in each group. Differences between the Control group and Treat 1 group and Treat 2 group in short-chain fatty acids are Propanoic acid, Butyric acid, Isovaleric acid and Valeric acid.

### Expression Analysis of Differential Metabolites

**Figure 3:**
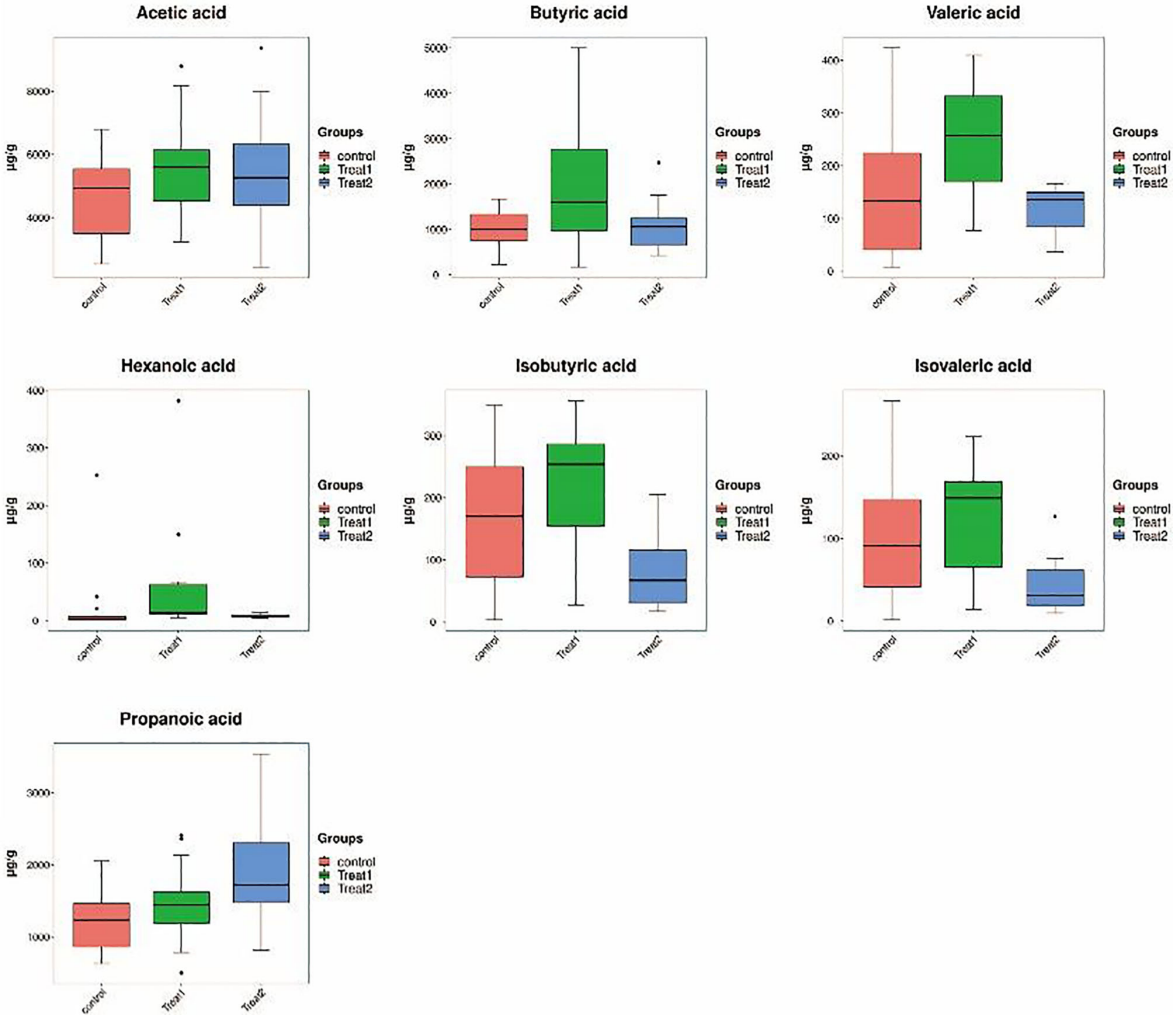
PCA plots of each group and differential expression heat maps of samples.

In order to further analyze the abundance differences of the 7 short-chain fatty acids detected in different groups, we visualized the abundance of the 7 short-chain fatty acids by histogram, and analyzed the significance of the differences between the 7 short-chain fatty acids in different groups by T test. The results showed that after GLP-1 treatment for type 2 diabetes, abundance of Butyric acid and Valeric acid in intestinal microbial metabolites was significantly up-regulated (p value <0.05). Propanoic acid and Isovaleric acid were significantly increased in intestinal microbial metabolites after treatment with probiotics for type 2 diabetes mellitus (p value <0.05). Compared with glP-1 treatment group, Isobutyric acid, Isovaleric acid and Valeric acid abundances of intestinal microbial metabolites were significantly up-regulated (p value <0.05) (**Figure 3**, **Table 2**).

**Table 2:** P value and FC details of short-chain fatty acids.

ID	Treat1-vs-control		Treat2-vs-control		Treat1-vs-Treat2	
	FC	P value	FC	P value	FC	P value
**Acetic acid**	1.203840	0.09477	1.161972	0.22002	1.036032	0.76456
**Propanoic acid**	1.179074	0.22029	1.537135	0.00858	0.767060	0.08591
**Isobutyric acid**	1.333119	0.17646	0.484263	0.01729	2.752880	0.00011
**Butyric acid**	1.952443	0.02271	1.069245	0.71578	1.826001	0.03553
**Isovaleric acid**	1.200805	0.47091	0.420870	0.01985	2.853153	0.00070
**Valeric acid**	1.626500	0.04049	0.750907	0.31120	2.166048	0.00029
**Hexanoic acid**	2.422759	0.27413	0.339449	0.361322	7.137335	0.07004

### Correlation Between Differential Metabolites and Differential Metabolites

**Figure 4:**
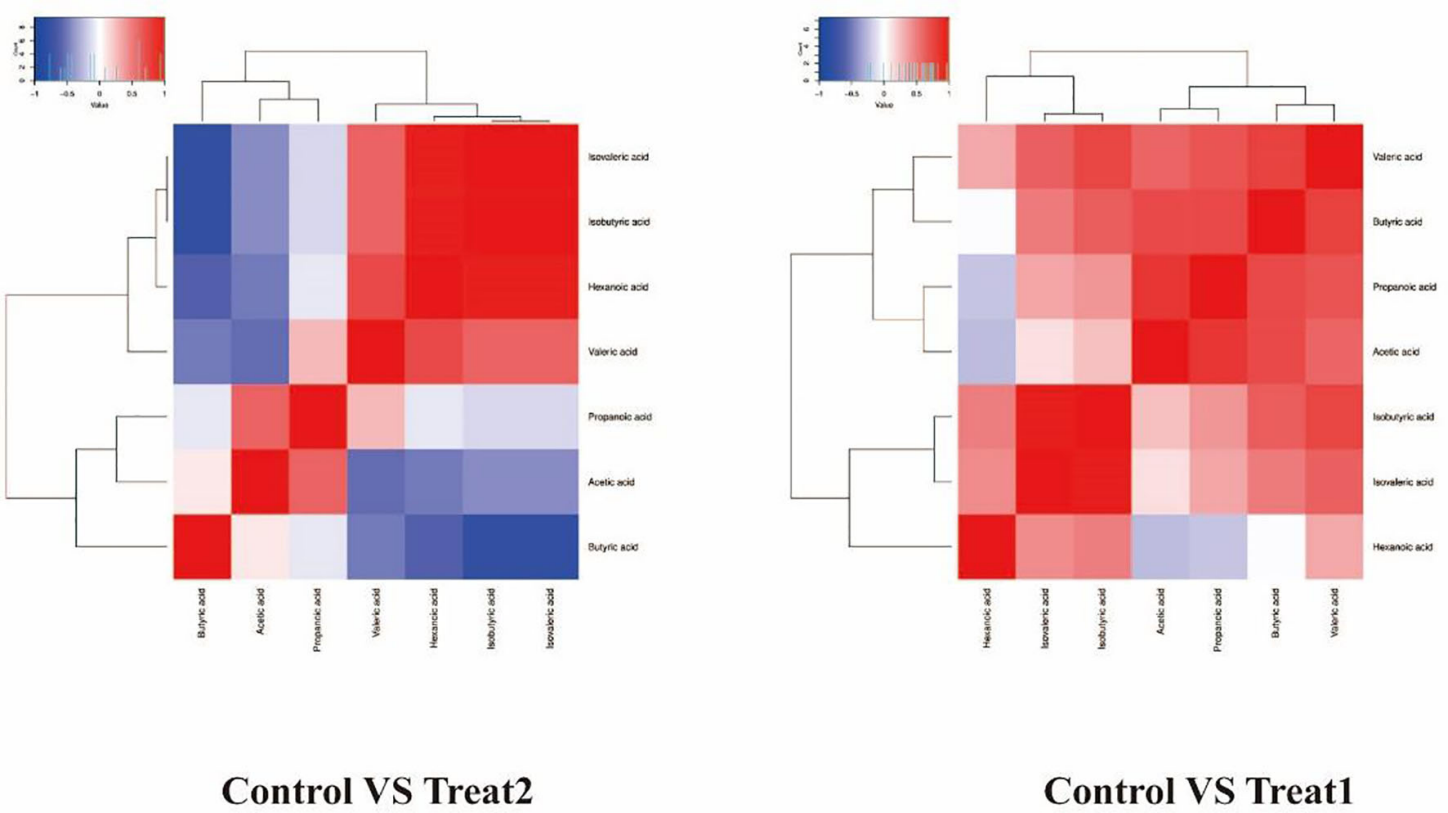
Metabolite association analysis.

In order to further analyze the relationship between differential short-chain fatty acids, we used the abundance data to conduct association analysis of 7 short-chain fatty acids between Treat 1 group and Treat 2 group and Control group, respectively. Results show that differential metabolites Butyric acid and Valeric acid are highly correlated after GLP-1 treatment for type 2 diabetes. The differential metabolite Propanoic acid after probiotic treatment of type 2 diabetes mellitus also had a high correlation with Isovaleric acid (**Figure 4**).

### KEGG Enrichment Analysis and Clinical Correlation Analysis of Metabolites

**Figure 5:**
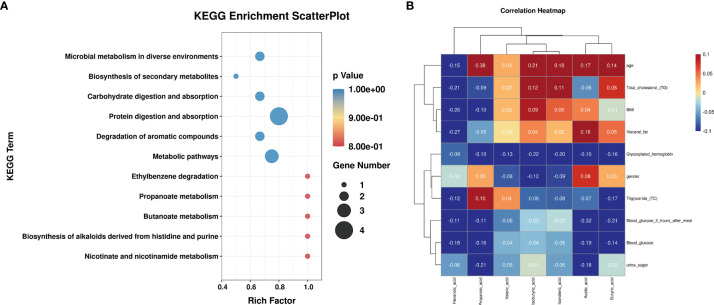
KEGG and clinical correlation analysis of metabolites. **(A)** is the KEGG signal pathway enrichment diagram of metabolites. **(B)** is the correlation analysis diagram between metabolites and clinical indicators.

In order to further study the main biological functions of differential metabolites after probiotics and GLP-1 treatment of type 2 diabetes, we conducted enrichment analysis of KEGG signal pathway for differential metabolites (**Figure 5A**, **Table 3**). The results showed that the differential metabolites were mainly concentrated in Protein Digestion and absorption and Metabolic pathways. Secondly, they were concentrated in mixed fermentation, Carbohydrate digestion and absorption and Microbial metabolism in diverse environments and Degradation of aromatic Come.

**Table 3:** Pathway corresponding to short-chain fatty acids.

pathway_id	pathway_name	Genes
760	Nicotinate and nicotinamide metabolism	Propanoic acid
1065	Biosynthesis of alkaloids derived from histidine and purine	Isovaleric acid
650	Butanoate metabolism	Butyric acid
640	Propanoate metabolism	Propanoic acid
642	Ethylbenzene degradation	Propanoic acid
1100	Metabolic pathways	Butyric acid, Propanoic acid, Isobutyric acid
1220	Degradation of aromatic compounds	Isobutyric acid, Propanoic acid
4974	Protein digestion and absorption	Butyric acid, Propanoic acid, Isobutyric acid, Isovaleric acid
4973	Carbohydrate digestion and absorption	Butyric acid, Propanoic acid
1110	Biosynthesis of secondary metabolites	Isovaleric acid
1120	Microbial metabolism in diverse environments	Propanoic acid, Isobutyric acid

Meanwhile, to explore the correlation between metabolite levels and clinical indicators, a correlation heat map was constructed (**Figure 5B**). The results showed that two different metabolites, Propanoic acid and Isovaleric acid, were negatively correlated with most diabetes-related indicators after probiotic treatment. Levels of Propanoic acid and Total cholesterol, BMI, Visceral fat, Glycosylated hemoglobin, Blood glucose 2 hours after meal, Blood Glucose and urine sugar were negatively correlated, but positively correlated with Triglyceride levels. Isovaleric acid was negatively correlated with Glycosylated hemoglobin, Triglyceride, Blood glucose 2 hours after meal, Blood glucose and urine sugar. Isovaleric acid was positively correlated with Total cholesterol, BMI and Visceral fat. Although differential short-chain fatty acids (Butyric acid and Valeric acid) are also correlated with diabetes-related indicators after GLP-1 treatment, as shown in **Figure 5B**, Except that Butyric acid is strongly correlated with Blood glucose 2 hours after meal and Triglyceride and Valeric acid is correlated with Glycosylated hemoglobin, The correlation between these two short-chain fatty acids and other indexes was weak.

## Discussion

In summary, we found that intestinal microbial short-chain fatty acid metabolism of patients with type 2 diabetes was changed after probiotics and GLP-1 treatment. Glp-1 treatment significantly increased Butyric acid and Valeric acid abundance in feces of patients (P<0.05), and the correlation between them was high. Butyric acid is the primary energy substrate of colon cells, stimulating the absorption of sodium and water in the colon and providing nutrition to intestinal cells (23). Current studies have shown that butyric acid can improve intestinal motility and has good therapeutic potential for inflammatory and functional intestinal diseases (24, 25). Meanwhile, Traisaeng et al. showed that butyric acid can reduce blood glucose and increase insulin content in type 1 diabetic mice, showing an anti-diabetic effect (26). Current studies have found that Valeric acid has anti-tumor effects. Han et al. found that Valeric acid has a broad spectrum of anti-tumor activities, showing particularly high cytotoxicity to liver cancer in cell proliferation, colony formation, wound healing, cell invasion and 3D spherogenesis (27). Shi et al. found that valerate may reduce breast cancer cell proliferation by mediating epigenetic modifications, such as inhibiting histone deacetylase and altering DNA methylation (28). Similarly, Valeric acid has the effect of alleviating intestinal radiation injury and protecting neuronal injury, and also has the regulation function of neurotransmitter (29–31). Therefore, the upregulation of Valeric acid after GLP-1 treatment seems to conform to the regulation effect of GLP-1 on the nervous system. In this study, it was found for the first time that GLP-1 not only regulates the blood glucose level of patients with type 2 diabetes, but also regulates the production of intestinal microbial metabolites butyric acid and Valeric acid, thus controlling the progression of diabetes from a multidimensional perspective. At the same time, through clinical follow-up studies, we found that both GLP-1 and probiotics treatment can improve the problem of hyperglycemia and hyperglycemia, and the proportion of patients with type 2 diabetes treated with probiotics guo returned to normal glucose levels was higher. Therefore, probiotics have great utility and potential in the treatment of type 2 diabetes.

Propanoic acid and Isovaleric acid were also associated with changes in the abundance of two short-chain fatty acids in the feces of patients with type 2 diabetes after probiotic treatment. Blakeney et al. showed that Isovaleric acid can directly act on colonic smooth muscle and cause muscle relaxation through the PKA pathway (32). At present, there are few studies on Propanoic acid and Isovaleric acid. Our study found that Propanoic acid plays a role in glucose metabolism homeostasis in the body. Probiotics can improve type 2 diabetes mellitus while changing the abundance of Propanoic acid and Isovaleric acid in intestinal microbial metabolites. Meanwhile, the abundance levels of Propanoic acid and Isovaleric acid are closely related, suggesting that Propanoic acid and Isovaleric acid have important roles. But it still needs to be validated in cells and animals.

Our study is the first to compare the glp-1 and probiotic preparations to treat type 2 diabetes after gut microbial metabolites of short chain fatty acids change, first of all, we found that in glp-1 and probiotics after treatment, there are large difference between the change of intestinal short chain fatty acids, this suggests glp-1 and in regulating intestinal probiotics treatment steady-state exist great differences, At present, it is not clear whether the changes of these short-chain fatty acids are beneficial to the body, and whether they regulate intestinal homeostasis or even metabolic homeostasis. Therefore, it is necessary to further verify the role of differential metabolites screened by us in regulating body energy and intestinal homeostasis in animal models. As microbial metabolic product - a product of the interaction between host and directly from dormitory or microbial biological processes, metabolic disease and is closely related with microorganism and metabolites, study of metabolites can make us to the current treatment method is used to have a deeper understanding, at the same time can provide potential target for the treatment of diseases.

KEGG signaling pathway enrichment indicated that different metabolites were associated with Protein Digestion, absorption and Metabolic pathways, and Digestion and absorption signaling pathways. These results suggest that the absorption of carbohydrate and protein (the two main nutrient species of the body) is regulated by probiotics after treatment of type 2 diabetes, which may be the way that probiotics treat type 2 diabetes by changing the balance of gut microflora and metabolism.

## Data Availability Statement

The original contributions presented in the study are included in the article/supplementary material. Further inquiries can be directed to the corresponding author.

## Author Contributions

QM and YW made equal contributions to this project. They jointly completed sample collection and data analysis of this project. TJ provided analytical thinking for this project. LZ, XW, YL, and YJW were responsible for clinical data collection and volunteer recruitment of the project, while NX was responsible for article writing and funding provision of the project. All authors contributed to the article and approved the submitted version.

## Funding

This project was supported by the Open Project of Clinical Medical Center of The First People’s Hospital of Yunnan Province (No. 2021LCZXXF-XY14) and the Open Project of Key Laboratory of Clinical Virology of Yunnan Province (No. 202005AG070062-016).

## Conflict of Interest

The authors declare that this study was conducted without any commercial relationship and that there is no potential conflict of interest with any institution or with the authors.

## Publisher’s Note

All claims expressed in this article are solely those of the authors and do not necessarily represent those of their affiliated organizations, or those of the publisher, the editors and the reviewers. Any product that may be evaluated in this article, or claim that may be made by its manufacturer, is not guaranteed or endorsed by the publisher.
